# Effect of dietary supplementation of rubber seed kernel pellet on feed utilization, rumen fermentation, fatty acid profiles and health status in swamp buffalo

**DOI:** 10.5713/ab.24.0354

**Published:** 2024-10-25

**Authors:** Nirawan Gunun, Chatchai Kaewpila, Waroon Khota, Thachawech Kimprasit, Pongsatorn Gunun

**Affiliations:** 1 Department of Animal Science, Faculty of Technology and Engineering, Udon Thani Rajabhat University, Udon Thani 41000, Thailand; 2 Department of Animal Science, Faculty of Natural Resources, Rajamangala University of Technology Isan, Sakon Nakhon 47160, Thailand; 3 Chakri Naruebodindra Medical Institute, Faculty of Medicine Ramathibodi Hospital, Mahidol Univerisity, Samut Prakan 10540, Thailand

**Keywords:** Fatty Acid Profiles, Feed Utilization, Health Status, Rubber Seed Kernel Pellet, Rumen Fermentation, Swamp Buffalo

## Abstract

**Objective:**

Rubber seed kernel is a by-product of rubber cultivation and are high in oil and protein. This study was conducted to evaluate the effect of supplementing rubber seed kernel pellet (RUSKEP) on feed intake, nutrient digestibility, rumen fermentation, rumen fatty acid profiles, blood parameters, and immune response in swamp buffalo.

**Methods:**

Four male swamp buffalo with an initial body weight (BW) of 254±10 kg and 26 months of age were used in this research. The experimental design was a 4×4 Latin squared design with RUSKEP supplementation at 0%, 4%, 6%, and 8% of dry matter intake (DMI). Animals were fed concentrate at 1.0% BW, while rice straw was fed *ad libitum*.

**Results:**

Supplementation with RUSKEP did not change DMI or nutrient digestibility (p>0.05), while ether extract digestibility increased cubically with the addition of RUSKEP (p<0.01). The ruminal pH, ammonia-nitrogen (NH_3_-N), and the proportion of acetate (C2) were similar among treatments (p>0.05). The proportion of propionate (C3) increased linearly (p≤0.04), leading to a decrease in the acetate to propionate ratio (C2:C3) (p≤0.04) with the addition of RUSKEP. Furthermore, the butyrate (C4) proportion decreased linearly with RUSKEP supplementation (p = 0.03). The addition of RUSKEP did not affect on linoleic acid (C18:2 cis-9,12+trans-9,12), or α-linolenic acid (C18:3 cis-9,12,15) (p>0.05). With RUSKEP supplementation, the stearic acid (C18:0) content increased quadratically (p<0.01). The increasing level of RUSKEP was higher in cholesterol and eosinophils (p≤0.03). The immune function (IgA, IgM, and IgG) was similar among treatments (p>0.05).

**Conclusion:**

Supplementing RUSKEP with up to 8% of DMI could improve rumen fermentation efficiency without affecting feed utilization, rumen PUFA profile, or immune response in swamp buffalo.

## INTRODUCTION

Smallholder farmers in Southeast Asia raise swamp buffalo (*Bubalus bubalis carabanesis*) for draft power and land cultivation through milk, meat, and fertilizer [1]. However, this animal’s role has transitioned from providing draft power to meat and milk production [2]. Swamp buffalo have a greater ability to metabolize fiber and produce a smaller amount of methane than cattle [3]. Most ruminants are fed low-quality roughage, particularly rice straw, which is low in crude protein (CP) and high in fiber content, which reduces the efficacy of ruminant production in tropical areas [4]. Therefore, supplementation of CP and energy can enhance animal feed efficiency, rumen fermentation, body weight (BW), and body condition score [5].

Southeast Asian regions, especially Thailand, primarily cultivate the rubber tree (*Hevea brasiliensis*). Rubber seed is a by-product of annual rubber tree production, estimated at 0.34 million metric tons per year [6]. Rubber seeds consist of a kernel and a shell, which account for approximately 60% and 40% of the total seed weight, respectively [7]. The rubber seed kernel (RSK) contains 20.1% to 22.2% CP, 33.4% to 38.0% ether extract (EE), and 7,834 to 8,031 kcal/kg dry matter (DM) gross energy [6,8]. The RSK has been considered a source of unsaturated fatty acids (UFA) primarily because of its composition of 22.5% oleic acid (OA; C18:1 cis-9), 40.0% linoleic acid (LA; C18:2 cis-9,12+trans-9,12), and 21.2% α-linolenic acid (ALA; C18:3 cis-9,12,15) [8]. According to our previous research, feeding yeast-fermented RSK at 10% to 25% had no impact on feed utilization, rumen fermentation characteristics, microbial population, microbial protein synthesis, or milk production in dairy cattle [6,9]. Gunun et al [8] reported that heating RSK in a hot air oven improves *in vitro* rumen fermentation by increasing the bacterial population and reducing ammonia-nitrogen (NH_3_-N), as well as reducing the biohydrogenation of ruminal UFA, particularly ALA and OA. Therefore, ruminants have the potential to utilize these by-products as protein and lipid sources.

Pelletizing has a variety of benefits for animal feeding, including preventing feed selection, increasing bulk and energy density, improving feed utilization, and rumen fermentation [10]. Moreover, pellets are convenient in terms of storage, transportation, and handling. In addition, fatty acids have the ability to regulate immune function and inflammatory processes [11]. Previous studies reported that the supplementation of oilseeds containing omega-3 (ω3) polyunsaturated fatty acids (PUFAs) can improve the immune responses of dairy cows [12]. Our previous studies revealed that the supplementation of rubber seed kernel pellet (RUSKEP) up to 10% enhanced *in vitro* rumen fermentation characteristics and concentration of C18 UFA, particularly ALA [13]. In this regard, the information gathered through *in vitro* trials must be confirmed *in vivo*. The impact of RUSKEP supplements on swamp buffalo digestion, rumen fermentation, rumen fatty acid profiles, and health status remains unexamined, and none of the existing investigations were performed at practical levels (4% to 8% dry matter intake [DMI]). We hypothesize that adding RUSKEP at suitable levels to ruminants could improve their rumen fermentation patterns, fatty acid compositions, and health status without having a negative effect on feed intake or nutrient digestibility. Therefore, the aim of this study was to investigate the effects of RUSKEP addition on feed intake, digestibility, rumen fermentation, rumen fatty acid profiles, blood parameters, and immune response in swamp buffalo.

## MATERIALS AND METHODS

### Animal care

The Animals Ethical Committee of Rajamangala University of Technology Isan approved all of the experimental animals and methodology used in this study (approval number 04-06-004).

### Preparation of RUSKEP

Rubber seeds were purchased from the local markets in Sakon Nakhon, Thailand, during the harvest season. The seeds were collected by hand from the ground and kept indoors. A dehulling machine (Incanewlife, Khon Kaen, Thailand) removed the shells from the seeds. The kernels were dried in sunlight for three days, followed by grinding to pass through a 1 mm sieve. The RUSKEP was prepared by using RSK as a major ingredient and other ingredients such as cassava starch, molasses, a mineral and vitamin mixture, or salt, and then making the mixture into pellets using a pellet machine.

### Animals, treatments, and experimental design

Four male swamp buffalo at 26 months of age and a BW of 254±10 kg were randomly assigned according to a 4×4 Latin square design. The buffalo were fed concentrate at 1% of the BW (Table 1) and rice straw *ad libitum*. The four dietary treatments were RUSKEP supplementation at 0%, 4%, 6%, and 8% of DMI. Animals were fed in two equal feeding times at 07:00 h and 16:00 h and provided with clean water. The buffalo were always kept in separate pens (3×4 m) with concrete floors and an iron fence. The experiment was conducted over four periods, each lasting 21 days. The feed adaptation period is the first 14 days, while the sample collection period is the final 7 days. A 7-day transition period occurred between each period.

### Data collection and sampling procedures

The feed that was offered and the refusals were recorded in the morning. The BW was measured daily during the sampling period, before feeding time. Samples of feed and feces were collected from every animal in the last 7 days of the period. The feces were collected from each animal to be used to evaluate their digestibility. Rectal sampling is used in collecting fresh feces samples (approximately 500 g) at 06:00 h and 15:00 h. The two successive samples have been combined and mixed before being stored in the refrigerator at 4°C. The samples, including concentrates, rice straw, RUSKEP, refusals, and feces, were dried at 60°C in a hot air oven and ground (1-millimeter screen using Cyclotech Mill; Tecator, Hoganas, Sweden). The DM, ash, and EE contents were analyzed following AOAC [14]. The CP content was determined using an N analyzer (828 Series; LECO, St. Joseph, MI, USA). The fiber analyzer (ANKOM 200; ANKOM Technology, Macedon, NY, USA) was used to determine the neutral detergent fiber (NDF) and acid detergent fiber (ADF) contents as described by Van Soest et al [15]. The fatty acid profiles in RUSKEP were determined using gas chromatography (GC 8890; Agilent Technologies Ltd., Santa Clara, CA, USA), according to Christie [16]. The nutrient digestibility was evaluated using acid-insoluble ash (AIA) as an internal marker [17].

On the final day of each period, a stomach tube connected to a vacuum pump collected approximately 200 mL of rumen fluid at 0 and 4 hours post-feeding. In order to prevent contact with saliva, the initial 100 mL of the ruminal samples were removed. The samples were followed by filtering via four layers of cheesecloth, and the pH was promptly determined with a portable pH meter (FiveGo; Mettler-Toledo GmbH, Greifensee, Switzerland). Ruminal fluid samples were centrifuged at 16,000×g for 15 minutes at 4°C, and the supernatant was kept at −20°C. The ruminal samples were analyzed for NH_3_-N (Kjeltech Auto 1030 Analyzer; Tecator) [18] and volatile fatty acid (VFA) using gas chromatography (Nexis GC-2030; Shizuku Co., Kyoto, Japan) [19]. The rumen fatty acid profiles were assessed using gas chromatography (GC 8890; Agilent Technologies Ltd.) with the Cristie [16] method.

Blood samples (approximately 10 mL) were obtained from the jugular vein concurrently with rumen fluid samples. Glucose, cholesterol, and total protein were evaluated with a chemical analyzer (Mindray BS-600; Mindray, Shenzhen, China). A hematology analyzer (Mindray BC-3000 Plus; Mindray) measured the blood’s hemoglobin, hematocrit, white blood cells (WBC), neutrophils, lymphocytes, monocytes, and eosinophils. Furthermore, the sampled blood was collected at 4 h post-feeding to evaluate immunoglobulins (IgA, IgG, and IgM) using the nephelometric technique (Mispa-i3; Agappe Diagnostics Ltd., Ernakulam, India).

### Statistical analysis

The data for variances were assessed using the general linear model in SAS software, using a 4×4 Latin square design [20]. The data were analyzed using the model *Yijk* = *μ*+*Mi*+ *Aj*+*Pk*+*ɛijk*, where *Yijk* = observation from treatment *i*; animal *j* and period *k*; *μ*, the overall mean; *Mi* = the mean effect of RUSKEP supplementation (*i* = 0%, 4%, 6%, 8%); *Aj* = the mean effect of animals (*j* = 1, 2, 3, 4); *Pk* = the mean effect of periods (*k* = 1, 2, 3, 4) and *ɛijk* the residual error. Orthogonal polynomial contrasts (linear, quadratic and cubic) were used to examine the effect of the levels of RUSKEP supplementation. At p<0.05, statistical significance was considered acceptable.

## RESULTS

### Chemical composition of diets

The concentrate can be formulated from local feed resources, which contain 14.2% CP, 1.0% EE, and 33.3% NDF (Table 1). The RUSKEP contains 17.1% CP and 34.2% EE (Table 2). In addition, the RUSKEP showed a higher UFA content, including OA, LA, and ALA.

### Feed intake and nutrient digestibility

The concentrate and rice straw intake were similar among treatments (p>0.05), while RUSKEP intake increased quadratically with increasing levels of RUSKEP (p≤0.02) (Table 3). The supplementation of RUSKEP did not alter the digestibilities of DM, OM, CP, NDF, and ADF (p>0.05). However, with the addition of RUSKEP, the EE digestibility increased cubically (p<0.01).

### Rumen fermentation

The ruminal pH and NH_3_-N concentration were similar among treatments (p>0.05) (Table 4). Adding RUSKEP increased cubically total VFA concentration at 4 h post-feeding (p = 0.03). The acetate (C2) proportion was not affected by the RUSKEP addition (p>0.05). The proportion of propionate (C3) increased linearly (p≤0.04), whereas C2:C3 decreased linearly (p≤0.04) at 0 and 4 h post-feeding with RUSKEP supplementation. Moreover, the butyrate (C4) decreased linearly at 4 h post-feeding with the increasing level of RUSKEP (p = 0.03).

### Rumen fatty acid profiles

Myristic acid (C14:0), pentadecanoic acid (C15:0), palmitic acid (C16:0), and heptadecanoic acid (C17:0) were lower with the addition of RUSKEP (p≤0.04) (Table 5). The RUSKEP supplementation increased quadratically stearic acid (C18:0) at 4 h post-feeding (p<0.01). The concentrations of OA, LA, and ALA were not affected when buffalo fed RUSKEP (p>0.05).

### Blood chemicals and hematological parameters

The addition of RUSKEP did not alter glucose or total protein (p>0.05) (Table 6). However, cholesterol increased quadratically at 0 and 4 h post-feeding with RUSKEP supplementation (p≤0.03). The hemoglobin, hematocrit, WBC, neutrophils, lymphocytes, and monocytes were similar among treatments (p>0.05), while eosinophils increased linearly with the addition of RUSKEP (p = 0.02).

### Immune response

Dietary supplementation of RUSKEP to buffalo had no effect on the concentrations of IgA, IgM, or IgG (p>0.05) (Table 7).

## DISCUSSION

The chemical composition of feedstuffs serves as a source of nutrients for animals. The CP and NDF content in RUSKEP was 17.1% and 26.5% in our study, which were similar to our previous research [13] using RUSKEP in an *in vitro* study. The higher EE content (34.2%) of RUSKEP was similar to our previous investigation, which found that the EE content of RSK ranged from 34.3% to 39.4% [6,9]. Moreover, the LA and ALA content of the RUSKEP were 43.7%, and 17.5%, respectively. Similarly, Gunun et al [13] reported that RUSKEP is high in PUFA and contains 49.7% LA and 13.4% ALA. Our previous research reported that RSK had 21.2% ALA [8]. The lower ALA content of RUSKEP than in previous studies may be due to the use of RSK as a major ingredient with other ingredients to make pellets, which could reduce the ALA content in the present study. The chemical composition of RUSKEP in this study suggests that buffalo can use it as a source of protein and lipids, particularly PUFA, in their diets.

Strategies for enhanced usage of animal feeds must be determined based on knowledge of their nutritional value, and the availability of feeds can be evaluated by feed utilization, rumen fermentation characteristics, blood parameters, as well as animal performance. The effects of unsaturated fat, including RSK supplementation, on DMI have been variable among studies. Our previous studies found that the inclusion of yeast-fermented RSK reduced roughage and total intake in dairy heifers [6]. Chanjula et al [21] observed that RSK supplementation in the diet reduced feed intake in goats. The addition of large amounts of fat to the ruminant may have a negative effect on palatability and lower feed intake [22]. In our study, however, adding RUSKEP had no effect on buffalo’s intake of concentrate and roughage. Similarly, Ouppamong et al [9] reported that adding yeast-fermented RSK to the diet did not affect feed intake in dairy cows. Moreover, RUSKEP supplementation enhanced the digestibility of EE. These results agree with our previous studies, which found that the inclusion of RSK in diets enhances EE digestibility in dairy cattle [6,9]. These findings could be attributed to the higher EE content of RUSKEP, which showed higher hydrolysis in the rumen compared to the control diet. Moreover, the addition of oilseeds is rich in UFA, which is toxic to ruminal bacteria, especially fibrolytic bacteria, which leads to lower fiber digestibility in animals [23]. Chanjula et al [21] reported that adding 30% RSK to the diet decreased fiber digestibility in goats. However, our previous studies found that adding yeast-fermented RSK up to 25% to the diet did not affect the fiber digestibility of dairy heifers [6]. In the current study, dietary supplementation with RUSKEP up to 8% of DMI had no effect on NDF and ADF digestibility in buffalo. This demonstrated that adding up to 8% of RUSKEP had no negative effect on feed intake or fiber digestibility in buffalo.

Rumen microorganisms ferment carbohydrates and lipids to produce VFA, which ruminants use as an energy source. Lipids in the diet, particularly tryglycerides, galactolipids, and phospholipids, are hydrolyzed by lipolytic bacteria and converted to glycerol, which subsequently ferments into VFA. Rumen microorganisms can convert glycerol into C2 and C3 [24]. The addition of RUSKEP at 6% and 8% of DMI increased total VFA concentration after feeding. The RSK consists of 34.2% EE, as found in our study, and 49.4% total carbohydrates [25]. After receiving the diets, the buffalo may convert the lipids and carbohydrates in the RUSKEP to high concentrations of C2 and C3, leading to an increase in the total VFA in the rumen. In addition, ruminal microbiota diversity, feed additives, and feed digestibility all have an impact on ruminal VFA proportion. There have been reports that oilseeds alter the proportions of VFA in the rumen [26]. The addition of RUSKEP enhanced the proportion of C3, leading to a reduction of C2:C3 in buffalo. These results agree with our previous studies, which found that supplementation with RUSKEP up to 10% increased the proportion of C3 and also had a lower C2:C3 *in vitro* [13]. Two possible mechanisms may account for the observed alteration in VFA proportion. First, rumen bacteria are still capable of degrading fatty acids in order to produce glycerol, as well as shifting to C3 [13]. Second, RSK contains higher soluble sugars [25], so adding it to diets might produce C3 in the rumen by microorganisms. Previous studies reported that ruminant fed high-starch and oilseed diets found to increase *Succinivibrio* species in the rumen are primarily responsible for increased C3 production [27,28], which possibility resulted in the reduced C2:C3 found in the current study. Furthermore, increasing levels of RUSKEP reduced the proportion of C4 in the rumen. These results are consistent with earlier research [29] using rapeseed, sunflower, and linseed oils to decrease C4 in the rumen. This suggests that butyrate-producing bacteria may be responsible for the LA toxicity effects in RUSKEP through metabolic pathways [30]. This study discovered that RUSKEP supplementation could improve rumen fermentation efficiency by increasing total VFA and propionate, resulting in reduced C2:C3 in the rumen. This possibility increases the availability of dietary energy, which in turn enhances the growth performance in buffalo.

The lipids in ruminant feedstuffs, especially oilseeds, are mainly C18 UFA, particularly OA, LA, and ALA. Upon entering the rumen, microorganisms are converted from C18 UFA to C18:0 via cis-trans isomerization to trans fatty acids, followed by hydrogenation of the double bonds; this is called biohydrogenation [31]. Nevertheless, the rumen’s reduction of C18 UFA to 18:0 is incomplete, resulting in an increased concentration of OA, LA, and ALA intermediates. Our previous research using an *in vitro* study showed that adding up to 10% RUSKEP increased OA, LA, and ALA as intermediates in the rumen [13]. In the current study, adding RUSKEP at 4% to 8% DMI did not alter the levels of OA. However, as RUSKEP levels increased, the concentration of LA and ALA decreased, although this difference was not statistically significant, leading to higher levels of C18:0 in the buffalo rumen. This indicated that the shift of C18 UFA in the RUSKEP to C18:0 completed the process of biohydrogenation by rumen microorganisms. The different results from our previous studies could be due to the differences between *in vitro* and *in vivo* studies. These variations might affect the roughage and concentrate feeding, as well as the community and activity of microbes in the rumen, resulting in a variation in the biohydrogenation of C18 UFA. The current study agrees with Abuelfatah et al [32] that the inclusion of whole linseed increased C18:0 without changing the OA or LA in the rumen of goats. Accorodingly, Tudisco et al [33] found significantly higher stearic acid content in milk of goats fed a diet higher in UFA levels, confiming the almost complete biohydrogenation of substrate in the rumen.

Cholesterol measurements can be used to determine the animal’s energy status. The higher levels of RUSKEP increased the concentration of cholesterol in the buffalo’s blood. Similarly, Pi et al [34] found that the addition of rubber seed oil increased blood cholesterol in dairy cows. Animals fed higher lipids in the diet may have enhanced fatty acid digestion and absorption, which may have led to the synthesis of cholesterol [35]. The ω3 PUFA, particularly eicosapentaenoic acid (EPA), has considerable potential to regulate inflammatory responses [36]. Eosinophils regulate inflammatory responses through lipid signaling [37]. Previous studies reported that the supplementation of fish oil enhanced the eosinophil numbers in calves [38]. Similarly, our study found that supplementation with RUSKEP increased eosinophil levels. Although this study does not assess the EPA in the blood, previous research found that the increased EPA in the cow’s blood was due to the rubber seed oil or linseed oil supplementation [39,40]. This could be PUFA feeding, which leads to increased levels of EPA in the blood and also increases eosinophils, according to our study.

The nutritional status and metabolism of nutrients in ruminants are essential for the correct functioning of the immune system and other cells. Dietary PUFA play an essential role in regulating the immunological response and inflammatory mediators. Previous research found that cows fed rubber seed oils and linseed oil enhanced the concentration of IgG but did not affect IgA and IgM [39]. In contrast with the current study, supplementation with PUFA rich in RUSKEP did not alter IgA, IgM, or IgG concentrations in buffalo. The concentration of immune function correlates with the levels of PUFA, particularly ALA, in the blood [38]. This indicated that adding up to 8% of RUSKEP was insufficient to alter the ALA and the immune response in the blood.

## CONCLUSION

Dietary supplementation with RUSKEP up to 8% enhanced rumen fermentation characteristics by increasing the concentration of total VFA and proportion of C3, while reducing C2:C3. Buffalo-fed RUSKEP regulates inflammatory functions by increasing eosinophils in the blood. However, it did not affect swamp buffalo’s feed utilization, rumen PUFA profile, or immune response.

## Figures and Tables

**Table 1:** Ingredients and chemical composition of the diet used in the experiment

Item	Concentrate	Rice straw
Ingredient (%, DM)		
Cassava chip	63.0	-
Soybean meal	16.5	-
Rice bran	15.0	-
Molasses	2.0	-
Urea	1.5	-
Mineral and vitamin mixture^1)^	1.0	-
Salt	0.5	-
Sulfur	0.5	-
Chemical composition		
Dry matter (%)	89.5	90.7
Organic matter (% DM)	88.4	85.0
Crude protein (% DM)	14.2	3.6
Ether extract (% DM)	1.0	0.3
Neutral detergent fiber (% DM)	33.3	73.2
Acid detergent fiber (% DM)	19.4	46.7
Ash (% DM)	11.6	15.0

1) Contains per kilogram premix: 10,000,000 IU vitamin A; 70,000 IU vitamin E; 1,600,000 IU vitamin D; 50 g Fe; 40 g Zn; 40 g Mn; 0.1 g Co; 10 g Cu; 0.1 g Se; 0.5 g I.

**Table 2:** Chemical compositions of rubber seed kernel pellet (RUSKEP)

Item	RUSKEP
Chemical composition	
Dry matter (%)	93.7
Organic matter (%DM)	95.0
Crude protein (%DM)	17.1
Ether extract (%DM)	34.2
Neutral detergent fiber (%DM)	26.5
Acid detergent fiber (%DM)	19.1
Ash (%DM)	5.0
Fatty acid (% of total fatty acid)	
C16:0	8.8
C16:1 cis-9	0.2
C18:0	7.1
C18:1 cis-9 (OA)	21.8
C18:2 cis-9,12+trans-9,12 (LA)	43.7
C18:3 cis-9,12,15 (ALA)	17.5
C20:0	0.2

OA, oleic acid; LA, linoleic acid; ALA, α-linolenic acid.

**Table 3:** Effect of rubber seed kernel pellet (RUSKEP) supplementation on feed intake and nutrient digestibility in swamp buffalo

Items	RUSKEP (% of DMI)				SEM	Contrast		
	
	0	4	6	8		L	Q	C
DMI								
Rice straw								
kg/d	4.1	4.1	3.8	4.0	0.28	0.67	0.78	0.44
%BW	1.4	1.5	1.4	1.5	0.08	0.80	0.88	0.32
Concentrate								
kg/d	2.9	2.7	2.6	2.7	0.09	0.15	0.45	0.92
%BW	1.0	1.0	1.0	1.0	0.01	0.12	1.00	0.19
RUSKEP								
kg/d	0.00	0.27	0.38	0.53	0.02	<0.01	0.02	0.05
%BW	0.00	0.10	0.14	0.20	0.05	<0.01	<0.01	0.02
Total intake								
kg/d	7.0	7.1	6.8	7.2	0.36	0.76	0.81	0.45
%BW	2.4	2.6	2.5	2.7	0.08	0.12	0.64	0.25
Digestibility coefficients (%)								
Dry matter	56.8	53.6	56.7	52.8	1.57	0.25	0.83	0.10
Organic matter	60.1	57.0	60.2	56.3	1.53	0.27	0.79	0.09
Crude protein	68.1	64.2	67.1	64.7	2.04	0.46	0.72	0.22
Ether extract	46.4	82.8	85.8	85.1	1.59	<0.01	<0.01	<0.01
Neutral detergent fiber	49.5	46.4	48.1	44.9	1.82	0.18	0.98	0.26
Acid detergent fiber	42.8	41.2	44.0	40.0	2.93	0.68	0.70	0.43

DMI, dry matter intake; SEM, standard error of the mean; L linear; Q, quadratic; C, cubic; BW, body weight.

**Table 4:** Effect of rubber seed kernel pellet (RUSKEP) supplementation on rumen fermentation in swamp buffalo

Items	RUSKEP (% of DMI)				SEM	Contrast		
	
	0	4	6	8		L	Q	C
Rumen pH								
0 h post-feeding	7.0	7.0	7.0	7.1	0.04	0.37	0.60	0.99
4 h post-feeding	6.8	6.9	6.8	7.0	0.04	0.09	0.34	0.25
NH_3_-N (mg/dL)								
0 h post-feeding	22.2	22.2	21.1	21.1	1.05	0.42	1.00	0.64
4 h post-feeding	23.4	22.2	25.7	22.2	1.34	0.99	0.41	0.10
Total VFA (mmoL/d)								
0 h post-feeding	47.6	69.1	61.8	66.8	8.10	0.21	0.34	0.30
4 h post-feeding	67.1	52.7	88.7	83.9	7.28	0.04	0.53	0.03
VFA (moL/100 moL)								
Acetate (C2)								
0 h post-feeding	75.1	75.8	71.2	71.6	1.69	0.09	0.93	0.21
4 h post-feeding	72.0	72.8	70.1	68.9	2.03	0.23	0.65	0.58
Propionate (C3)								
0 h post-feeding	15.2	15.4	19.5	21.3	1.40	0.01	0.57	0.36
4 h post-feeding	17.9	16.7	20.8	24.3	2.0	0.04	0.28	0.53
Butyrate (C4)								
0 h post-feeding	9.7	8.8	9.3	7.1	0.74	0.06	0.39	0.25
4 h post-feeding	10.1	10.5	9.1	6.8	0.93	0.03	0.19	0.86
C2:C3								
0 h post-feeding	5.0	5.0	3.8	3.4	0.40	0.01	0.68	0.28
4 h post-feeding	4.1	4.4	3.4	3.0	0.40	0.04	0.46	0.35

DMI, dry matter intake; SEM, standard error of the mean; L linear; Q, quadratic; C, cubic; VFA, volatile fatty acid.

**Table 5:** Effect of rubber seed kernel pellet (RUSKEP) supplementation on rumen fatty acid profile in swamp buffalo

Items	RUSKEP (% of DMI)				SEM	Contrast		
	
	0	4	6	8		L	Q	C
Fatty acid (% of total fatty acid)								
C10:0								
0 h post-feeding	1.7	0.1	0.4	0.3	0.61	0.19	0.24	0.43
4 h post-feeding	0.1	0.5	0.1	0.1	0.18	0.66	0.48	0.22
C12:0								
0 h post-feeding	1.9	0.6	1.4	1.3	0.52	0.67	0.30	0.25
4 h post-feeding	1.0	0.6	1.5	2.0	0.18	0.18	0.47	0.55
C13:0								
0 h post-feeding	0.5	0.3	0.2	0.3	0.14	0.36	0.46	0.85
4 h post-feeding	0.5	0.4	0.1	0.3	0.06	0.16	0.21	0.21
C14:0								
0 h post-feeding	6.8	2.8	4.3	2.4	1.04	0.04	0.36	0.11
4 h post-feeding	5.2	2.2	2.5	3.1	0.57	0.06	0.02	0.26
C14:1 cis-9								
0 h post-feeding	1.1	0.4	0.2	1.2	0.46	0.92	0.09	0.76
4 h post-feeding	1.4	0.2	0.3	0.3	0.59	0.27	0.36	0.63
C15:0								
0 h post-feeding	4.7	2.6	1.5	2.5	0.27	<0.01	<0.01	0.48
4 h post-feeding	5.2	2.2	2.1	2.8	0.37	<0.01	<0.01	0.23
C16:0								
0 h post-feeding	29.9	20.8	27.2	21.1	3.22	0.21	0.65	0.10
4 h post-feeding	34.0	21.1	23.9	20.8	1.32	<0.01	<0.01	0.01
C16:1 cis-9								
0 h post-feeding	0.3	0.1	0.4	0.4	0.15	0.34	0.40	0.24
4 h post-feeding	0.5	0.1	0.2	0.6	0.17	0.55	0.06	0.59
C17:0								
0 h post-feeding	1.8	1.1	0.8	0.8	0.30	0.06	0.29	0.97
4 h post-feeding	2.4	0.8	0.5	0.7	0.41	0.02	0.07	0.63
C18:0								
0 h post-feeding	31.9	62.8	51.9	54.5	6.07	0.08	0.06	0.09
4 h post-feeding	36.3	55.4	55.9	56.1	2.33	<0.01	<0.01	0.13
C18:1 cis-9 (OA)								
0 h post-feeding	5.1	5.4	8.6	7.2	2.19	0.36	0.71	0.47
4 h post-feeding	6.0	10.5	9.8	9.2	1.77	0.29	0.20	0.53
C18:2 cis-9,12+trans-9,12 (LA)								
0 h post-feeding	2.2	1.4	1.3	1.8	0.59	0.60	0.34	0.95
4 h post-feeding	2.8	3.0	1.6	1.6	0.50	0.06	0.86	0.22
C18:3 cis-9,12,15 (ALA)								
0 h post-feeding	0.3	0.5	0.4	0.3	0.17	0.70	0.48	0.84
4 h post-feeding	1.6	0.7	0.4	0.7	0.79	0.42	0.47	1.00
C20:0								
0 h post-feeding	2.3	1.0	0.8	0.6	0.64	0.11	0.37	0.73
4 h post-feeding	0.9	0.7	0.9	1.0	0.33	0.63	0.58	0.78

DMI, dry matter intake; SEM, standard error of the mean; L linear; Q, quadratic; C, cubic; OA, oleic acid; LA, linoleic acid; ALA, α-linolenic acid.

**Table 6:** Effect of rubber seed kernel pellet (RUSKEP) supplementation on blood chemistry and hematology in swamp buffalo

Items	RUSKEP (% of DMI)				SEM	Contrast		
	
	0	4	6	8		L	Q	C
Glucose (mg%)								
0 h post-feeding	74.3	71.0	72.5	73.5	3.67	0.96	0.58	0.75
4 h post-feeding	80.5	81.3	77.5	76.8	4.01	0.43	0.85	0.69
Cholesterol (mg/dL)								
0 h post-feeding	47.3	59.8	64.0	63.8	2.27	<0.01	0.03	0.72
4 h post-feeding	45.0	58.8	63.3	62.3	1.62	<0.01	<0.01	0.62
Total protein (g/dL)								
0 h post-feeding	7.1	7.0	6.9	6.9	0.07	0.12	0.20	0.65
4 h post-feeding	7.1	6.9	6.8	6.7	0.15	0.06	0.45	0.53
Hemoglobin (g/dL)								
0 h post-feeding	15.4	12.8	12.6	12.3	0.89	0.05	0.24	0.55
4 h post-feeding	13.0	12.2	11.8	13.5	0.97	0.79	0.25	0.70
Hematocrit (%)								
0 h post-feeding	46.5	38.7	38.0	37.0	2.68	0.05	0.25	0.56
4 h post-feeding	39.3	37.0	35.8	40.8	2.93	0.81	0.26	0.70
White blood cells (10^9^/L)								
0 h post-feeding	11.5	12.3	11.3	10.8	0.55	0.25	0.27	0.39
4 h post-feeding	13.2	12.7	13.0	12.3	1.07	0.64	0.91	0.76
Neutrophils (%)								
0 h post-feeding	44.5	47.0	38.0	45.0	3.09	0.60	0.49	0.09
4 h post-feeding	48.8	43.5	44.5	45.8	2.67	0.52	0.26	0.63
Lymphocytes (%)								
0 h post-feeding	45.8	43.5	50.5	41.8	2.64	0.68	0.26	0.07
4 h post-feeding	43.0	47.0	45.0	41.0	3.48	0.62	0.29	0.80
Monocytes (%)								
0 h post-feeding	4.3	4.8	5.0	3.8	0.61	0.66	0.20	0.66
4 h post-feeding	5.0	4.8	4.5	5.5	0.64	0.67	0.36	0.67
Eosinophils (%)								
0 h post-feeding	3.0	2.0	5.0	6.0	0.93	0.02	0.32	0.20
4 h post-feeding	1.8	2.3	3.5	5.3	0.92	0.02	0.52	0.95

DMI, dry matter intake; SEM, standard error of the mean; L linear; Q, quadratic; C, cubic.

**Table 7:** Effect of rubber seed kernel pellet (RUSKEP) supplementation on immune response in swamp buffalo

Items (mg/dL)	RUSKEP (% of DMI)				SEM	Contrast		
	
	0	4	6	8		L	Q	C
IgA	160.8	163.5	170.3	163.5	5.38	0.56	0.41	0.49
IgM	72.0	73.5	68.8	72.3	1.93	0.66	0.62	0.14
IgG	632.8	658.0	670.8	653.8	11.13	0.18	0.10	0.74

DMI, dry matter intake; SEM, standard error of the mean; L linear; Q, quadratic; C, cubic; IgA, immunoglobulin A; IgM, immunoglobulin M; IgG, immunoglobulin G.
